# Mental Disorders Among Health Care Workers at the Early Phase of COVID-19 Pandemic in Kenya; Findings of an Online Descriptive Survey

**DOI:** 10.3389/fpsyt.2021.665611

**Published:** 2021-07-22

**Authors:** Edith Kamaru Kwobah, Ann Mwangi, Kirtika Patel, Thomas Mwogi, Robert Kiptoo, Lukoye Atwoli

**Affiliations:** ^1^Department of Mental Health, Moi Teaching and Referral Hospital, Eldoret, Kenya; ^2^Department of Mathematics, Physics and Computing, Moi University, Eldoret, Kenya; ^3^Department of Immunology, Moi University, Eldoret, Kenya; ^4^Directorate of Informatics, Moi Teaching and Referral Hospital, Eldoret, Kenya; ^5^Department of Medicine, Aga Khan University Medical College, East Africa, Nairobi, Kenya; ^6^Department of Mental Health and Behavioral Sciences, Moi University, Eldoret, Kenya

**Keywords:** prevalence, health care workers, Kenya, mental disorders, COVID-19

## Abstract

**Background:** Healthcare workers responding to the Corona Virus Pandemic (COVID-19) are at risk of mental illness. Data is scanty on the burden of mental disorders among Kenyan healthcare workers responding to the pandemic that can inform mental health and psychosocial support. The purpose of this study was to establish the frequency and associated factors of worry, generalized anxiety disorder, depression, posttraumatic stress disorder and poor quality of sleep among Kenyan health care workers at the beginning of COVID-19 pandemic.

**Methods:** We conducted an online survey among 1,259 health care workers in Kenya. A researcher developed social demographic questionnaire and several standardized tools were used for data collection. Standardized tools were programmed into Redcap, (Research Electronic Data Capture) and data analysis was performed using R Core Team. In all analysis a *p*-value < 0.05 was considered significant.

**Results:** 66% of the participants reported experiencing worry related to COVID-19. 32.1% had depression, 36% had generalized anxiety, 24.2% had insomnia and 64.7% scored positively for probable Post Traumatic Stress Disorder (PTSD). Depression was higher among females compared to men (36.5 vs. 26.9%, *p* = 0.003), workers <35 years old compared to older ones (38.1 vs. 26.4%, *p* < 0.001), and those who were not married compared to those who were married (40.6 vs. 27.6%, *p* < 0.001). Generalized anxiety was commoner among workers aged <35 years (43.5 vs. 29.3%, *p* < 0.001), females (41.7 vs. 29.2%, *p* < 0.001), those who mere not married compared to the married (45.2 vs. 31.2%, *p* < 0.001) and those with <10 years working experience (41.6 to 20.5%, *p* < 0.001). Younger health care professional had a higher proportion of insomnia compared to the older ones (30.3 vs. 18.6%, *p* < 0.001). Insomnia was higher among those with <10 years' experience compared to those with more than 20 years' experience(27.3 vs. 17.6%, *p* = 0.043)

**Conclusion:** Many Kenyan healthcare workers in the early phase of COVID-19 pandemic suffered from various common mental disorders with young, female professionals who are not married bearing the bigger burden. This data is useful in informing interventions to promote mental and psychosocial wellbeing among Kenyan healthcare workers responding to the pandemic.

## Background

The mental health impact of the Corona Virus disease of 2019 (COVID-19) is expected be huge due to the nature of the disease and the containment measures imposed (1). There is sufficient evidence to indicate that during any epidemic or pandemic, healthcare workers are affected mentally to a great extent (2). Unique to the case of COVID-19 pandemic in the early phase was the widespread media coverage, high rates of human to human transmission, and the fact that a lot is still unknown about this new virus, which may all contribute to poor mental health among these health care professionals. The ever-increasing number of confirmed and suspected cases, overwhelming workload, depletion of personal protection equipment, lack of specific curative drugs, and healthcare workers feelings of being inadequately supported significantly contribute to the risk of their mental health illness (3).

An increase in mental and psychological distress during epidemics has been documented in literature. As an example, the heath care workers who responded to the 2014-2015 Ebola epidemic in Africa reported high levels of psychological symptoms (4). Similarly, during the outbreak of Severe Acute Respiratory Syndrome (SARS) in Hong Kong and Beijing, 20% of the total number of reported cases in the end were of frontline healthcare workers, who had to face a tremendous mental burden in addition to physical strain in taking care of patients with the highly contagious disease (5). Further, a previous study found that during the outbreak of infectious diseases, the affected hospitals experienced severe staff shortage as a result of personal or family health concerns, child care issues, quarantine measures or inability to get to work, and that health care workers were particularly worried for both their own and their family's health, and they experienced significant psychosocial trauma (6). In Singapore following the SARS outbreak, doctors had to be more vigilant when examining and reviewing patients, and it is reported that 20% of the doctors and nurses suffered PTSD -Post Traumatic Stress Disorder (7).

During a pandemic, healthcare workers are likely to express worry, besides fulfilling criteria for specific mental health disorders. A rapid review of literature done between March and April 2020 reported that health care workers working during the current pandemic reported frequent worry regarding their own health and the fear of infecting their families, friends and colleagues (8). A web based survey that included 31 countries across the globe, including Kenya, conducted between April and May 2021 across the globe reported that 60% of health care workers had anxiety and 53% had mild to moderate depression (9). A review of the immediate impact of Covid-19 revealed that up to 35% of healthcare workers are likely to be report symptoms of traumatic stress (10).

There has been a call across the globe to put in all possible measures to preserve and enhance resilience of healthcare workers (11) and for managers to proactively take steps to protect the mental well-being of staff (12). Such efforts need to be backed by evidence of the burden of the mental illness among these front liners, but unfortunately there is scanty data from the Kenyan context and other low resource settings to guide interventions to improve mental health and psychological support among healthcare workers. Such evidence would be useful not only for Kenya but for similar contexts in low and middle income countries where human resource for health is especially constrained, hence are likely to be stretched some more by the current pandemic, thus increasing their risk of mental ill health (13). This study therefore sought to 1. Determine the Frequency of worry, generalized anxiety disorder, depression, posttraumatic stress disorder (PTSD) and poor quality of sleep among health care professionals at the beginning of the COVID-19 pandemic- [these conditions were selected as they have been demonstrated as being highly prevalent among healthcare workers even before the pandemic (14, 15)], and 2.To establish the association between socio-demographic characteristics and mental disorders among health care professionals at the beginning of COVID-19 pandemic in Kenya.

## Materials and Methods

A cross- sectional descriptive online survey was used for this study. Trained healthcare professionals working in healthcare settings during the COVID-19 19 pandemic were included—these included, nurses, doctors, clinical officers and public health officers interacting with patients at the time of the study. Trained nurses, doctors, clinical officers and public health officers who do not interact with patients on day to day basis such as professionals working with insurance companies, those working in administration settings only or those who work exclusively in teaching institutions were excluded.

The survey questionnaires were programmed into Redcap, (Research Electronic Data Capture) a secure, web-based software platform designed to support data capture for research studies (16). A researcher developed social demographic questionnaire was used to collect data on age, sex, cadre, place of work, role during the pandemic and years of experience. Standardized tools were used for data collection. For anxiety we used Generalized Anxiety Disorder 7 (GAD 7) which we scored 0–4 = no anxiety, 5–9 = mild anxiety, 10–14 = moderate anxiety and ≥15- severe anxiety (17). GAD 7 has demonstrated good internal consistency and convergent validity in heterogeneous samples (18) and has been used within Kenyan settings (19, 20). For depression we used Patient Health Questionnaire (PHQ 9) for depression which we scored 0–5 = Mild depression, 6–10 = moderate depression, 11–15 = moderately severe and 16–20 =severe depression (21). PHQ 9 is a widely used tool for screening for depression with a good reliability and convergent validity (22) and has been used previously in Kenya, having demonstrated good content validity in a study in Western Kenya (23). For PTSD we used Primary Care- Post traumatic Stress disorder (PC- PTSD) for Diagnostic Statistical Manual (DSM) V—those who responded positively to 3 out of 5 questions about how the traumatic event(s) had affected them over the past month was regarded as probable PTSD (24). Though this has not been validated in a Kenyan context it has been shown to have high diagnostic accuracy and is highly acceptable due to its simplicity and brevity- having only five questions (25) and has shown good reliability even outside the US setting, as is the case of the Korean validation (26). We used the term Probable PTSD because to make a diagnosis of PTSD we would need a more comprehensive assessment by a clinician. For sleep difficulties we used Pittsburg Sleep Quality Index (PSQI), a self-rated questionnaire which assesses sleep quality and disturbances over a 1-month time interval, and used the following cut-offs; 0–7 = no clinically significant insomnia; 8–14 = subthreshold insomnia; 15–21 = clinical insomnia of moderate severity; 21–28 = severe clinical insomnia (27). Though not validated for use in Kenya it has been validated for other African settings such as Ethiopia (28) and Nigeria (29).

We used the Cochran formulae for sample size calculation for a survey, assuming a 50% prevalence of common mental disorders and a 95% confidence level: sample Size = 1.96^2^ *[0.5 × (1–0.5)]/[(0.05^2^)]. The minimum sample for each of the cadres was 384 (30). To allow for incomplete data, we planned to recruit at least 400 participants for each of the cadres.

A virtual snowball convenient sampling technique was utilized to recruit participants given. This was necessitated by limitation of a clear database for healthcare workers' contacts which would allow randomization. The online survey was sent to different healthcare workers in various networks on Facebook, WhatsApp and emails. A weekly reminder requesting workers to participate was sent between April 27^th^ and June 5^th^ 2020. The healthcare workers were requested to respond to the survey and share with their colleagues while a track of responses was kept using the Redcap software until there were no new responses for a period of 2 weeks, at which point we closed the survey.

Descriptive statistics were used to summarize the socio demographic characteristics of the participants. Chi square test was used in the bivariate analysis to assess categorical factors associated with the various mental health disorders and significant variables at 0.20 were considered in the multivariable logistic regression analysis and presented as adjusted odds ratios (AORs) with the corresponding 95% Confidence Intervals. The variables considered in the regression analysis were the various social demographics characteristics (age, gender, years of experience, cadre), having existing medical conditions, having contacts with a confirmed COVID-19 patient and the type of facility one was working in. Data analysis was performed using R Core Team (2013). In all analysis a *p*-value < 0.05 was considered significant.

## Results

### Socio Demographic Characteristics

Though we initially hoped for 2,000 participants (400 per cadre for 5 cadres) only a total of 1,259 participants opened the survey after 6 weeks of sharing the link. A total of 69 did not consent to participate in the study. A further 233 consented but didn't respond to the various sections of the questionnaire including the socio demographic and were thus excluded from the analysis. Nine hundred fifty seven participants completed at least one or more components of the questionnaire, giving a response rate of 76%.

The median age of the participants was 35 years (IQR: 30–42). Majority 522 (54.5%) were females, married 619 (64.7%), non-specialist doctors 378 (39.5%), Christians 794 (83.0%) had an undergraduate level of education 433 (45.5) and had <10 years of experience in the medical field 546 (57%). Majority were working in a public facility 672 (70.2%) and most were in level 6 facility 306 (32%). Most of the participants reported that they did not have a medical condition 739 (77.2%) and also that they had never been treated for a mental illness 739 (77.2%) (Table 1).

**Table 1:** Sociodemographic characteristics of the participants.

	**(*N* = 957)**
**Age (yrs)**	
Mean (SD)	37.39 (10.18)
Range	20.00 - 83.0
**Gender**	
Male	435 (45.5%)
Female	522 (54.5%)
**Marital status**	
Married	619 (64.7%)
Single	272 (28.4%)
Widowed/Separated/Divorced	47 (4.9%)
Other	19 (2.0%)
**Religion**	
Muslim	118 (12.3%)
Christian	794 (83.0%)
Other	45 (4.7%)
**Cadre**	
Doctor non-specialist	378 (39.5%)
Doctor specialist	156 (16.3%)
Nurse	186 (19.5%)
Other	236 (24.7%)
**Education level**	
Diploma	151 (15.8%)
Higher diploma	59 (6.2%)
Undergraduate	435 (45.5%)
Masters	270 (28.2%)
Phd/Fellowship	42 (4.4%)
**Years of experience**	
0–10	546 (57.0%)
11–20	239 (25.0%)
20+	108 (18.0%)
**Facility**	
Public	672 (70.2%)
Private	285 (29.8%)
**Level of facility**	
Level 6 National	306 (32.0%)
Level 5 county referral	273 (28.5%)
Level 4 Subcounty	217 (22.7%)
Level 3 Heath Center	112 (11.7%)
Level 2 Dispensary	49 (5.1%)
**Have a known medical condition**	
Yes	218 (22.8%)
No	739 (77.2%)
**Ever been treated for mental illness**	
Yes	58 (6.1%)
No	899 (93.9%)
**Currently working directly with COVID-19 clients**	
Yes	231 (24.1%)
No	726 (75.9%)

### Frequency and Factors Associated With Mental Disorders

#### Worry

Overall, 66% of the participants were “Quite a bit” or “Very worried” about at least one of the various issues related to COVID-19. Participants reported being “Quite a bit” or “Very worried” about: contracting COVID-19 (71%) being hospitalized for COVID-19 (58%), dying of COVID-19 (43%), losing a loved one due to COVID-19 (66%), being rejected due to COVID-19 (42%), infecting others with COVID-19 (65%) and not being able to do what they know best (61%). At least a third of the HCWs were worried about each of the COVID-19 related issues, including contacting the disease, dying and infecting others.

Being worried about COVID-19 19 was less likely to be reported among participants who had more years of experience compared to those who had less year (64 vs. 69%, *p* = 0.026), specialist doctors compared to other cadres (56 vs. 73% *p* = 0.006) and those who were working in private facilities compared to working in public facilities (61 vs. 68 % *p* = 0.024). Those who had contact with COVID-19 patients had higher likelihood of reporting worry than those who had not had contact (71 vs. 64%, *p* = 0.040) (Table 2).

**Table 2:** Factors associated with Worry among Healthcare workers.

**Variable**	**Levels**	**Worry (*****N*** **= 957)**		
		**Not very much (*n* = 322)**	**Very much (*n* = 635)**	***P*-value**
Age in years	<35	135 (29.2)	327 (70.8)	0.262
	≥35	187 (37.8)	308 (62.2)	
Sex	Male	155 (35.6%)	280 (64.4%)	0.235
	Female	167 (32.0%)	355 (68.0%)	
Marital status	Married	214 (34.6%)	405 (65.4%)	0.828
	Not married	86 (31.6%)	186 (68.4%)	
Years of experience	0–10	165 (30.2%)	381 (69.8%)	0.026
	11–20	95 (39.7%)	144 (60.3%)	
	20+	62 (36.0%)	110 (64.0%)	
Cadre	Specialist	68 (43.6%)	88 (56.4%)	0.006
	Doctor	132 (34.9%)	246 (65.1%)	
	Nurse	59 (31.7%)	127 (68.3%)	
	Other	63 (26.7%)	173 (73.3%)	
Facility	Public	211 (31.4%)	461 (68.6%)	0.024
	Private	111 (38.9%)	174 (61.1%)	
Have known medical condition	Yes	67 (30.7%)	151 (69.3%)	0.300
	No	255 (34.5%)	484 (65.5%)	
Contact COVID-1919 clients	Yes	65 (28.1%)	166 (71.9%)	0.040
	No	257 (35.4%)	469 (64.6%)	

Being worried about COVID-19 19 was less likely to be reported among participants who had more years of experience compared to those who had less year (64 vs. 69%, *p* = 0.026), specialist doctors compared to other cadres (56 vs. 73% *p* = 0.006) and those who were working in private facilities compared to working in public facilities (61 vs. 68 % *p* = 0.024). Those who had contact with COVID-19 patients had higher likelihood of reporting worry than those who had not had contact (71 vs. 64%, *p* = 0.040) (Table 2).

#### Depression

One third of the participants 283/599 (32.1%) scored positively for depression, with a majority having moderate depression. On bivariate analysis, age, sex, marital status and years of experiences were significantly associated with depression. A higher proportion of female (36.5%) had moderate/sever symptoms of depression compared to males (26.9%), *p* = 0.003. In terms of age there was a higher proportion with depression among those <35 years (38.1%) compared to those above 35 years (26.4%), *p* < 0.001. Heath care providers who were married reported lower rates of depression (27.6%) compared to those who were not married (40.6%), *p* < 0.001. While the proportion with depression decreased as the number of years of experience in the medical increased; 37% for those with <10 years and 20% for those with above 20 years of experience, *p* < 0.001 (Table 3).

**Table 3:** Factors associated with mental disorders on bivariate analysis.

**Variable**	**Levels**	**PHQ (*****N*** **= 882)**			**GAD (*****N*** **= 807)**			**PSQI (*****N*** **= 780)**		
		**Mild**	**Moderate/severe**	***P*-value**	**Present**	**None**	***P*-value**	**Insomnia**	**No insomnia**	***P*-value**
Age in years	<35	265 (61.9)	163 (38.1)	<0.001	167 (43.5)	217 (56.5)	<0.001	114 (30.3)	262 (69.7)	<0.001
	≥35	334 (73.6)	120 (26.4)		124 (29.3)	299 (70.7)		75 (18.6)	329 (81.4)	
Sex	Male	296 (73.1)	109 (26.9)	0.003	106 (29.2)	257 (70.8)	<0.001	73 (20.8)	278 (79.2)	0.052
	Female	303 (63.5)	174 (36.5)		185 (41.7)	259 (58.3)		116 (27.0)	313 (73.0)	
Marital status	Married	419 (72.4)	160 (27.6)	<0.001	165 (31.2)	363 (68.8)	<0.001	110 (21.9)	392 (78.1)	0.052
	Not married	180 (59.4)	123 (40.6)		126 (45.2)	153 (54.8)		79 (28.4)	199 (71.6)	
Years of experience	0-10	320 (62.9)	189 (37.1)	<0.001	191 (41.6)	268 (58.4)	<0.001	123 (27.3)	327 (72.7)	0.043
	11–20	155 (71.4)	62 (28.6)		70 (34.7)	132 (65.3)		42 (21.6)	152 (78.4)	
	20+	124 (79.5)	32 (20.5)		30 (20.5)	116 (79.5)		24 (17.6)	112 (82.4)	
Cadre	Specialist	106 (70.7)	44 (29.3)	0.454	40 (28.4)	101 (71.6)	0.013	24 (17.5)	113 (82.5)	0.022
	Doctor	241 (67.7)	115 (32.3)		116 (36.8)	199 (63.2)		80 (25.5)	234 (74.5)	
	Nurse	116 (70.7)	48 (29.3)		47 (30.9)	105 (69.1)		27 (19.4)	112 (80.6)	
	Other	135 (64.0)	76 (36.0)		87 (43.9)	111 (56.1)		58 (30.7)	131 (69.3)	
Facility	Public	426 (69.4)	188 (30.6)	0.182	190 (33.7)	373 (66.3)	0.046	133 (24.8)	404 (75.2)	0.668
	Private	173 (64.6)	95 (35.4)		101 (41.4)	143 (58.6)		56 (23.0)	187 (77.0)	
Have known medical condition	Yes	138 (69.7)	60 (30.3)	0.600	73 (39.0)	114 (61.0)	0.378	50 (28.6)	125 (71.4)	0.155
	No	461 (67.4)	223 (32.6)		218 (35.2)	402 (64.8)		139 (23.0)	466 (77.0)	
Contact COVID-1919 clients	Yes	141 (68.1)	66 (31.9)	1.000	76 (39.8)	115 (60.2)	0.253	47 (25.1)	140 (74.9)	0.816
	No	458 (67.9)	217 (32.1)		215 (34.9)	401 (65.1)		142 (23.9)	451 (76.1)	

Adjusting for age, years of experience, cadre and type of facility on a multivariate logistic regression, sex and marital status were statistically significantly associated with depression. The odds of moderate/severe depression was 1.5 times in females compared to males (AOR = 1.47; 95% CI: 1.09, 2.00, *p* = 0.012). The odds of depression in health workers who were not married was 1.5 times that of those who were married (AOR = 1.47; 95%CI: 1.07, 2.01, *p* = 0.016). HCWS with more years of experience were less likely to score positively for depression (AOR 0.50, 95% CI 0.28–0.88, *p* = 0.016) (Table 4).

**Table 4:** Multivariate logistic regression.

**Variable**	**PTSD**			**GAD**			**PSQI**		
	**AOR**	**95% CI**	***P*-value**	**AOR**	**95% CI**	***P*-value**	**AOR**	**95% CI**	***P*-value**
**Age in years** <35	1			1			1		
≥35	0.83	0.53, 1.28	0.400	0.76	0.47, 1.20	0.200	0.49	0.28, 0.83	0.010
**Sex** Male	1			1			1		
Female	1.47	1.09, 2.00	0.012	1.72	1.25, 2.37	<0.001	1.39	0.98, 1.99	0.067
**Marital status** Married	1			1			1		
Not married	1.47	1.07, 2.01	0.016	1.41	1.02, 1.96	0.036	1.15	0.80, 1.64	0.500
**Years of experience** 0–10	1			1			1		
11–20	0.75	0.46, 1.21	0.200	0.92	0.56, 1.52	0.700	1.27	0.70, 2.33	0.400
20+	0.50	0.28, 0.88	0.016	0.49	0.27, 0.90	0.021	1.12	0.55, 2.28	0.800
**Cadre** Specialist	1			1			1		
Doctor	0.70	0.43, 1.14	0.150	0.94	0.57, 1.56	0.800	1.20	0.68, 2.17	0.500
Nurse	0.85	0.51, 1.43	0.500	1.01	0.59, 1.74	0.900	0.92	0.49, 1.73	0.800
Other	1.04	0.64, 1.70	0.900	1.59	0.96, 2.63	0.072	1.72	0.98, 3.10	0.064
**Facility** Public	1			1					
Private	1.29	0.94, 1.78	0.120	1.57	1.13, 2.20	0.008	-		
**Known Medical condition**									
Yes							1		
No	-						0.66	0.45, 0.99	0.045
**Contact COVID-19 clients**									
Yes			1						
No			0.75	0.53, 1.07	0.110	-			

### Generalized Anxiety Disorder

About a third of the participants, 291/807 (36%) scored positively for generalized anxiety. Those who experienced sleeping difficulties were 24.2% (189/780). On bivariate analysis, age, sex, marital status, years of experience in medical profession and cadre were statistically significantly associated with generalized anxiety disorder. Younger health care profession reported a higher proportion with generalized anxiety (43.5%) compared to those aged more than 35 years (29.3%), *p* < 0.001. Females had a higher proportion with generalized anxiety compared to males (41.7 vs. 29.2%), *p* < 0.001, while married had a lower proportion compared to those who were not married (31.2 vs. 45.2%), *p* < 0.001. In terms of experience as the number of years of experience in the health profession increased from <10 years to more than 20 years the proportion with generalized anxiety decreased from 41.6 to 20.5% *p* < 0.001, and the Doctor specialist reported the lowest proportions of generalized anxiety (28.4%) compared to other workers; non-specialist doctors 36.8%, nurses 30.9, and other cadres 43.9 %, *p* = 0.013 (Table 3).

Adjusting for age, years of experience, cadre and having contact with COVID-19 patients' on a multivariate logistic regression analysis, factors associated with generalized anxiety were sex and, marital status and the type of facility were associated with generalized anxiety. Females had a higher odds of having anxiety compared to males (AOR = 1.72; 95%CI: 1.25, 2.37, *p* = <0.001). Not being married and working in private facility increased the odds of having generalized anxiety (AOR = 1.41; 95%CI: 1.02, 1.96, *p* = 0.036), and (AOR = 1.57; 95%CI: 1.13, 2.20, *p* = 0.008), respectively. HCWS with more years of experience were less likely to score positively for GAD (AOR 0.49, 95% CI 0.27–0.97, *p* =0021) (Table 4).

### Insomnia (Pittsburg Sleep Quality Index)

15 /957 (1.9%) of the healthcare workers had clinically moderate insomnia while 174/ 957 (22.3%) had subthreshold insomnia. On bivariate analysis, age, cadre and years of experience in the health profession were statistically significantly associated with insomnia. Younger health care professional had a higher proportion with insomnia (30.3%) compared to 18.6% among those over 35 years old, *p* < 0.001. Proportion with Insomnia was higher among those with less than 10 years' experience (27.3%) compared to those with more than 20 years' experience in the health profession (17.6), *p* = 0.043. Other cadres had higher proportions of insomnia (30.7) compared to specialist doctors (17.5), *p* = 0.022 (Table 3).

Adjusting for sex, marital status, years of experience,cadre and having a known medical condition in a multivariate logistic regression, health professionals aged 35 years and above had a reduced odds of reporting insomnia compared to the younger ones (AOR = 0.49; 95%CI: 0.28,0.83) (Table 4).

### PTSD

For PTSD majority of the health workers didn't respond to the questions. Of the ones who responded 225/348 (64.7%) had some probable PTSD (Table 5). Due to the few number of people responding to the trauma questions, we did not do further analysis on this item.

**Table 5:** Frequency of various mental disorders among Kenyan Healthcare workers.

	**Overall (*N* = 957)**
**Depression (PHQ)**	
N-Miss	75
Mild	599 (67.9%)
Moderate	147 (16.7%)
Moderately severe	93 (10.5%)
Severe	43 (4.9%)
**Generalized Anxiety Disorder**	
N-Miss	150
None	516 (63.9%)
Mild	163 (20.2%)
Moderate	69 (8.6%)
Severe	59 (7.3%)
**PC-PTSD (DSMV)**	
N-Miss	609
None	123 (35.3%)
Probable PTSD	225 (64.7%)
**Pittsburg Sleep Quality Index**	
N-Miss	177
No Insomnia	591 (75.8%)
Clinically moderate Insomnia	15 (1.9%)
Subthreshold Insomnia	174 (22.3%)

## Discussions

This study adds to the existing literature on the frequency of mental illness among health care workers responding to COVID-19 in Kenya. We found a substantial degree of worry related to COVID-19 among healthcare workers in the early phase of the pandemic. The frequency of depression, anxiety, insomnia and probable PTSD were high. Young female workers with less experience in the healthcare profession were more likely to score positively for mental illness.

### Worry

In our study two thirds of the participants were “Quite a bit” or “Very worried” about at least one of the various issues related to COVID-19. The leading three issues participants worried about were losing a loved one to COVID-19, infecting others, and getting infected with COVID-19. While worry may not be classified as a mental illness, it is of importance as it could be a precursor of more severe mental illness if not addressed (31). These findings are comparable to those of a Japanese study that showed that majority (78%) of the healthcare workers were seriously worried about COVID-19 and being infected was one of the leading concerns (32). The high frequency of worry in our setting is in keeping with a recent review that indicated that other than the main diagnostic categories of mental illness, many healthcare workers have various concerns regarding COVID-19 that increase their risk of experiencing psychological distress (33). Participants who had more years of experience, were specialist doctors, or were working in private facilities had lower likelihood of reporting worry, while those who had had contact with COVID-19 patients had higher likelihood of reporting worry. Such worry has been associated with the fact that this disease is new, is highly contagious, has no cure and there is rapidly evolving information about its outcomes (34). This is also likely due to the several reports of healthcare workers contracting the disease and a good number succumbing to it in various part of the world (35).

### Depression

Our study established high rates of depression among healthcare workers; 15.4% having moderately severe to severe depression and 16% having moderate depression. Our findings differ from a similar study done by Onchonga et al. in the same setting which reported that 53% of healthcare workers had mild depression while 9.2% had severe depression (36). This difference may be attributed to the timing of data collection during the pandemic as different waves may present with different levels of psychological distress. These findings are higher than that of a similar study involving 150 healthcare workers from Nepal which reported that 5.3% had moderate depression while 2.7% had moderately severe to severe depression (37). A study done among 906 healthcare workers from Singapore and India reported that 5.3% had moderate to severe depression (38). Our findings are also higher than a recent systematic review and meta-analysis done during the COVID-19 season that included 13 studies with total of 33,062 participants reported a pooled prevalence of 22.8% for depression, although it is not clear the cut offs for depression that were used (39). These differences may be attributed to the differences in the settings of these studies and the social demographic characteristics of the participants.

Several factors were associated with Depression in our study. A higher proportion of female had moderate/severe symptoms of depression compared to males. This is similar to findings of a nationwide study done in India among 433 healthcare workers that reported that women were twice likely to have depressive symptoms requiring treatment (40). This may be attributed to the fact that even in the general population, females are at a higher risk of depression owing to various psychological and cultural factors (41). A study done in Chinese healthcare workers also found being male to be protective for depression (42). Health care providers who were married reported lower rates of depression compared to those who were not married and this is likely because marriage provides a support system that reduces the risk of depression. There was a higher proportion with depression among those <35 years compared to those above 35 years and the proportion with depression decreased as the number of years of experience in the medical field increased. This is in line with a study done among South African doctors that demonstrated that junior doctors are likely to experience burnout and depression and this could partly be attributed to the resilience that is built over years of practice resulting in less emotional exhaustion (43).

### Anxiety

In our study, a third of the participants (36%) had generalized anxiety. This is higher than the recent meta-analytic evidence which reported a pooled prevalence of 23% (39). These findings are comparable to those of the Nepal study that reported that 34% had anxiety but differed among those who had severe anxiety in Nepal where only 2% had moderately severe to severe anxiety (37).

Younger health care profession reported a higher proportion of generalized anxiety compared to those aged more than 35 years. This is in agreement with a Chinese study which also showed higher rates of anxiety among the younger healthcare workers compared to the older ones (44). Females had a higher proportion with generalized anxiety compared to males. This is in agreement with A Turkish study which indicated that females were at higher risks of generalized and health related anxiety compared to their male counterparts (45). These findings may be because, female gender has been associated with anxiety even in the general population. Those who were married had a lower proportion of anxiety compared to those who were not married. This may be explained by the social support that marriage brings to an individual. In terms of experience as the number of years of experience in the health profession increased from <10 years to more than 20 years the proportion with generalized anxiety decreased from and the doctor specialist reported the lowest proportion with generalized anxiety and these two may be associated with the confidence that comes with practice and managing more complex patients.

### Sleeping Difficulties

Upto 24.2% of the healthcare workers reported sleeping difficulties. This is comparable to findings of a study done in China including both the heath care workers and the general population which reported that of the 5641 respondents, 20% had clinically significant insomnia (46). It was also higher than that of an Ethiopian study that reported a prevalence of 12.4%, but lower than the pooled prevalence of insomnia among healthcare workers from a meta-analytic evidence which was reported to be 38.9% (39).

In our study, younger health care professionals had a higher proportion with insomnia compared to those over 35 years old. Proportion with Insomnia decreased with increased experience in the health profession and those in other cadres other than nurses and doctors had a higher proportion with insomnia. Sleep difficulties among healthcare workers during this pandemic are important as they may not only increase vulnerability to the virus but also affect productivity of the workers who need to be at their optimal performance as they combat the virus (47).

### Probable PTSD

It is worth noting that many participants did not respond to the trauma question. The reason for this is not clear and would warrant further evaluation of the understanding of trauma concepts in the Kenyan setting. Of those who responded, two thirds had been exposed to potentially traumatic events that could result in posttraumatic stress disorder. This is slightly higher than findings of a study done among 863 medical care workers from seven provinces in China using the Impact of Event Scale-6 which reported a prevalence of 40.2% (48). Similar high rates of PTSD have been shown in previous Flu out breaks. For example a study done among nurses working in south Korea during the Middle East Respiratory Syndrome (MERS) reported that more than half of the develop PTSD (49). The higher rates may be explained by the high mortality rates associated with Covid-19 being published throughout the globe that increases the risk of vicarious trauma among the providers.

### Relevance to Policy and Practice

This study provides an evidence base for the burden of mental illness among healthcare workers responding to COVID-19 pandemic in Kenya. The burden which is likely to worsen as the pandemic lingers on is likely to further compromise the healthcare systems which were already understaffed prior to the pandemic. This evidence put together is a call to prioritize Kenyan healthcare worker wellness by putting in all possible measures to preserve and enhance resilience of healthcare workers and to link to care those who are affected in order to sustain productivity and ensure continuity.

A global survey involving 32 countries already reported that healthcare workers are employing various coping strategies to cope such as positive thinking and getting family support (50). Government as well as institutional efforts to help healthcare workers implement these coping strategies in order to enable them continue working with COVID-19 patients are highly recommended. These efforts include managers taking proactive steps to protect the mental well-being of staff, to be frank about the challenging situations, and to allow teams to come together often to discuss their experiences and check on each other's well-being as well as link to care those who actually develop mental illness.

## Study Limitations

While these findings are novel, a few limitations must be born in interpreting these findings. First this was an online survey and may have response bias where by the non-responders might be more stressed health care professionals who did not respond to the online survey. Second Online surveys are less accessible to some groups of people (the older professionals especially) hence creating a bias. Third, cross-sectional data can identify associations, not causal relationships with COVID-19. Fourth we used convenient sampling hence the results may not be generalizable to other settings. Finally, there are several personal factors that could influence mental health of individuals that were not considered in this study. Nevertheless our study provides more evidence for the burden of mental ill health among healthcare workers that would inform efforts to promote mental well-ness among healthcare workers during the current pandemic and in future similar situations.

## Conclusions

This study established that there is a high frequency of depression, anxiety, insomnia and probable PTSD among healthcare workers responding to COVID-19 pandemic in Kenya with the highest burden being among young, less experienced female professionals. This evidence is a call to all relevant authorities to put in place measures to enhance healthcare workers mental and psychosocial well-ness in order to enable healthcare workers participate optimally in the fight against COVID-19. Further, longitudinal studies are recommended to better understand the causal relationship between COVID-19 and mental ill health among healthcare workers in Kenya and similar settings.

## Data Availability Statement

The original contributions presented in the study are included in the article/Supplementary Material, further inquiries can be directed to the corresponding author/s.

## Ethics Statement

The studies involving human participants were reviewed and approved by Moi University/MTRH Institutional Research and Ethics Committee. The ethics committee waived the requirement of written informed consent for participation.

## Author Contributions

KP and EK developed the initial concept. EK, LA, KP, TM, AM, and RK were involved in concept development, review of manuscript, and approval for final submission. AM did the statistical analysis. TM programmed the survey into Redcap and performed data cleaning. LA sourced for funding. All authors contributed to the article and approved the submitted version.

## Conflict of Interest

The authors declare that the research was conducted in the absence of any commercial or financial relationships that could be construed as a potential conflict of interest.

## Abbreviations

AOR: adjusted odds ratio
COVID-19: corona virus disease of 2019
DSMV: diagnostic statistical manual version V
GAD: generalized anxiety disorder
PHQ: patient health questionnaire
PTSD: post-traumatic stress disorder.
