# Supplementary material for: Mental Disorders Among Health Care Workers at the Early Phase of COVID-19 Pandemic in Kenya; Findings of an Online Descriptive Survey
Source: Front Psychiatry. 2021 Jul 22;12:665611. doi: 10.3389/fpsyt.2021.665611 (PMC8339368; doi:10.3389/fpsyt.2021.665611)
Supplement: Supplementary file 1 [file Table_1.DOCX]

**Additional Tables**

Prevalence of various types of worry

|  | Overall (N=957) |
| --- | --- |
| **Contracting COVID** |  |
| Never | 42 (4.4%) |
| A little | 231 (24.1%) |
| Quite a bit | 321 (33.5%) |
| Very much | 363 (37.9%) |
| **Being hospitalized for COVID** |  |
| Never | 162 (16.9%) |
| A little | 242 (25.3%) |
| Quite a bit | 214 (22.4%) |
| Very much | 339 (35.4%) |
| **Dying of COVID** |  |
| Never | 271 (28.3%) |
| A little | 271 (28.3%) |
| Quite a bit | 129 (13.5%) |
| Very much | 286 (29.9%) |
| **Losing a loved one due to COVID** |  |
| Never | 126 (13.2%) |
| A little | 201 (21.0%) |
| Quite a bit | 232 (24.2%) |
| Very much | 398 (41.6%) |
| **Being Rejected due to COVID** |  |
| Never | 302 (31.6%) |
| A little | 252 (26.3%) |
| Quite a bit | 190 (19.9%) |
| Very much | 213 (22.3%) |
| **Infecting others with COVID** |  |
| Never | 135 (14.1%) |
| A little | 200 (20.9%) |
| Quite a bit | 251 (26.2%) |
| Very much | 371 (38.8%) |
| **Not being able to do what you know best** |  |
| Never | 158 (16.5%) |
| A little | 211 (22.0%) |
| Quite a bit | 273 (28.5%) |
| Very much | 315 (32.9%) |

Worry by background characteristics of respondents:

| **Variable** | **Levels** | **Worry (N=957)** | | |
| --- | --- | --- | --- | --- |
|  |  | Not very much  (n=322) | Very much (n=635) | **p-value** |
| **Age in years** | <35 | 135 (29.2) | 327 (70.8) | 0.262 |
|  | >=35 | 187 (37.8) | 308 (62.2) |  |
| **Sex** | Male | 155 (35.6%) | 280 (64.4%) | 0.235 |
|  | Female | 167 (32.0%) | 355 (68.0%) |  |
| **Marital status** | Married | 214 (34.6%) | 405 (65.4%) | 0.828 |
|  | Not married | 86 (31.6%) | 186 (68.4%) |  |
| **Years of experience** | 0-10 | 165 (30.2%) | 381 (69.8%) | 0.026 |
|  | 11–20 | 95 (39.7%) | 144 (60.3%) |  |
|  | 20+ | 62 (36.0%) | 110 (64.0%) |  |
| **Cadre** | Specialist | 68 (43.6%) | 88 (56.4%) | 0.006 |
|  | Doctor | 132 (34.9%) | 246 (65.1%) |  |
|  | Nurse | 59 (31.7%) | 127 (68.3%) |  |
|  | Other | 63 (26.7%) | 173 (73.3%) |  |

| **Facility** | Public | 211 (31.4%) | 461 (68.6%) | 0.024 |
| --- | --- | --- | --- | --- |
|  | Private | 111 (38.9%) | 174 (61.1%) |  |
| **Have known medical condition** | Yes | 67 (30.7%) | 151 (69.3%) | 0.300 |
|  | No | 255 (34.5%) | 484 (65.5%) |  |
| **Contact COVID19 clients** | Yes | 65 (28.1%) | 166 (71.9%) | 0.040 |
|  | No | 257 (35.4%) | 469 (64.6%) |  |
